# Call for papers on plant breeding for complex traits

**DOI:** 10.1093/g3journal/jkag255

**Published:** 2026-09-25

In February 2023, the journals of the Genetics Society of America—GENETICS and G3: Genes|Genomes|Genetics—launched the series on Plant Genetics and Genomics to showcase papers on genetic and functional discovery in plants. Today, we announce the formation of a complementary series on **Plant Breeding for Complex Traits**, to highlight the development and application of new methods for crop and ornamental plant improvement. Complex, polygenic traits remain the primary targets and greatest challenges for plant breeding.

While not an exhaustive list, we invite high-quality original research on such topics as the following:

- Integrating biological knowledge for genomic prediction, such as evolutionary constraint or physiology
- Leveraging environomics and phenomics for genetic gain
- Using image analysis to select on plant architecture and product appearance
- Heritability of high-throughput phenotyping
- Optimization and simulation methods for breeding program design
- Genome editing techniques and applications to complex traits

Series Editors:

- Jeffrey Endelman, University of Wisconsin-Madison, United States
- James Holland, US Department of Agriculture, United States
- Kabin Xie, Huazhong Agricultural University, China

Authors are invited to submit manuscripts to GENETICS or G3: Genes|Genomes|Genetics by 2027 January 18. Manuscripts will be peer reviewed and edited according to the standards of the GSA Journals. Manuscripts submitted to GENETICS may be offered a transfer to G3 or vice versa.

Articles will appear online via Advance Access shortly after acceptance and will be initially published in an issue of GENETICS and G3 in Fall 2027. Papers will be promoted in a collection that is discoverable across the two journals and will be promoted via social media and other venues in a manner similar to other series the journals have published.

We encourage continued submissions past 2027 January 18, as this series will be an ongoing and growing resource in this topical area. At submission, please choose “Plant Breeding for Complex Traits” in the submission system and mention the series in your cover letter.

Please share this call with your colleagues and contact any of the series editors or the GENETICS and G3: Genes|Genomes|Genetics Editorial Office with questions, suggestions, or presubmission inquiries.

